# [Retracted] SFRP2 modulates non‑small cell lung cancer A549 cell apoptosis and metastasis by regulating mitochondrial fission via Wnt pathways

**DOI:** 10.3892/mmr.2024.13305

**Published:** 2024-08-12

**Authors:** Peng Li, Shu Zhao, Yi Hu

Mol Med Rep 20: 1925–1932, 2019; DOI: 10.3892/mmr.2019.10393

Following the publication of this paper, it was drawn to the Editor's attention by a concerned reader that certain of the JC1-stained cellular images shown in Fig. 2C on p. 1928 were strikingly similar to data that had already been published in different form in another article written by different authors at different research institutes [Yao S and Yan W: Overexpression of Mst1 reduces gastric cancer cell viability by repressing the AMPK-Sirt3 pathway and activating mitochondrial fission. Onco Targets Ther 11: 8465-8479, 2019].

Owing to the fact that the contentious data in the above article had already been published prior to its submission to *Molecular Medicine Reports*, the Editor has decided that this paper should be retracted from the Journal. The authors were asked for an explanation to account for these concerns, but the Editorial Office did not receive a satisfactory reply. The Editor apologizes to the readership for any inconvenience caused.

